# Immune reconstitution inflammatory syndrome mediated maxillary mucocele: a rare presentation

**DOI:** 10.1016/j.bjorl.2024.101495

**Published:** 2024-08-31

**Authors:** Vishwanath Gowda, Elsa Babu, Rajesh Radhakrishna Havaldar, Shilpa Mallapur

**Affiliations:** Jawaharlal Nehru Medical College, Department of ENT and HNS, Kaher, Belagavi, Karnataka, India

## Introduction

Mucoceles develop as a result of fluid accumulation when there is obstruction to the drainage of normal sinus secretion. This obstruction can be attributed to past facial trauma, previous surgery or chronic rhinosinusitis. Most commonly seen are frontoethmoidal mucoceles, whereas maxillary and sphenoid sinus mucoceles are rare.

Immune Reconstitution Inflammatory Syndrome (IRIS) occurs in approximately 10%–25% of patients with HIV who receive highly active antiretroviral therapy (HAART).^2^

The goal of HAART is immune restoration, resulting in a decrease of opportunistic infections. However, this immune restoration can be associated with a paradoxical clinical deterioration, due to an exaggerated immune-inflammatory reaction against live or dead pathogens. Such a paradoxical reaction is called Immune Reconstitution Inflammatory Syndrome (IRIS).^1^

An abundance of mast cells is known to be found in the nasopharyngeal connective tissue of HIV-infected individuals. IRIS encompasses a variety of conditions that occur temporally with CD4 increases caused by HAART. IRIS is labelled by clinical, or laboratory parameters paradoxically worsened despite 2–4 fold CD4 count increase during the first 12 months of HAART.

The time interval between the initiation of HAART and the evolution of IRIS ranges from less than 1 week to several months. Most cases of IRIS occur during the first 8 weeks of HAART and develop among patients with CD4 less than 100 cells/mm^3^.^2^

Patients with HIV have decreased mucociliary clearance of their sinuses, with predisposition to obstruction of the sinus ostia and microbial overgrowth. HIV also causes an allergic diathesis with increased serum Immunoglobulin E (IgE) levels and allergic reactivity.^2^

There is a significant correlation between serum IgE levels and sinus severity, suggesting that sinusitis may be part of the acquired atopic state that has been reported in patients with HIV. Furthermore, among HIV-positive patients with sinusitis and/or atopy, IgE levels are higher at CD4 less than 200 cells/mm^3^.^2^

## Case report

A 44-year-old male patient, HIV seropositive since 6 months presented to ENT OPD with complaints of purulent discharge from right nasal cavity for 3 months. He also had history of frontal headache since 3 months. History of hyposmia was present. The patient denied nasal obstruction, trauma, facial pain, fever, visual disturbance or diplopia.

He was started on Highly Active Antiretroviral Therapy (HAART) since 6 months Dolutegravir (50 mg), Lamivudine (300 mg) and Tenofovir Disoproxil Fumarate (300 mg), and cotrimoxazole one tablet per day; prescribed by a local physician. His CD4 cell count was 50 cells/mm^3^ before antiretrovirals were initiated. Over the course of 20 weeks of HAART, the CD4 count had increased to 136 cells/mm^3^.

The patient was afebrile with no other comorbidities.

**Figure 1:**
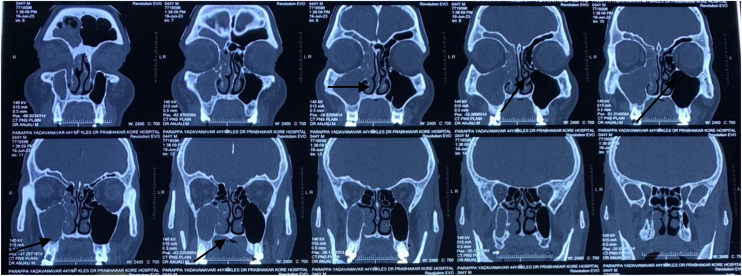
CT PNS plain.

Diagnostic nasal endoscopy showed left high deviated nasal septum with posterior spur, right nasal cavity showed hypertrophied inferior turbinate and accessory maxillary ostia. However plain CT paranasal sinuses (Fig. 1) showed features suggestive of sinonasal polyposis resulting in obliteration of osteomeatal complex, frontoethmoidal recess and nasolacrimal duct on the right side. The patient was initially given a course of oral antibiotics and antihistamines for a week before hospital admission. Patient was not prescribed oral or topical steroids due to presence of HIV.

Hypodense mucosal thickening with soft tissue pacification noted involving the right maxillary sinus with extension into ipsilateral nasal cavity via widened osteomeatal complex resulting in the complete obliteration of sinus, nasal cavity, osteomeatal complex and nasolacrimal duct on right side. Hypodense mucosal thickening with soft tissue pacification noted involving right frontal and right ethmoidal sinus with obliteration of right frontoethmoidal recess.

**Figure 2:**
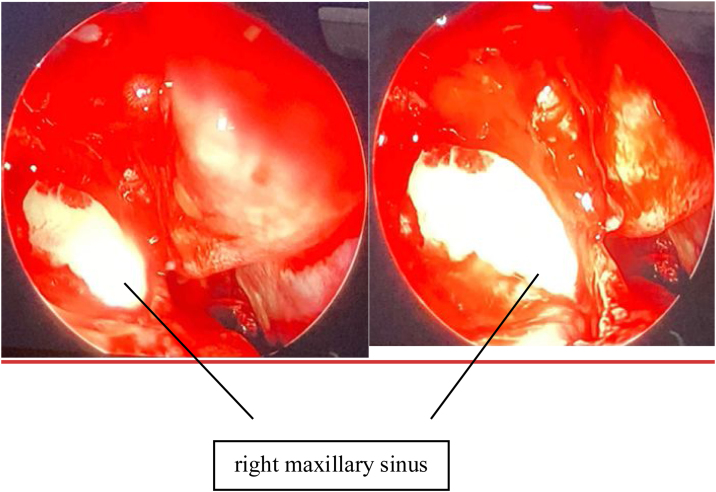
Intraoperative endoscopic view of right maxillary sinus.

**Figure 3:**
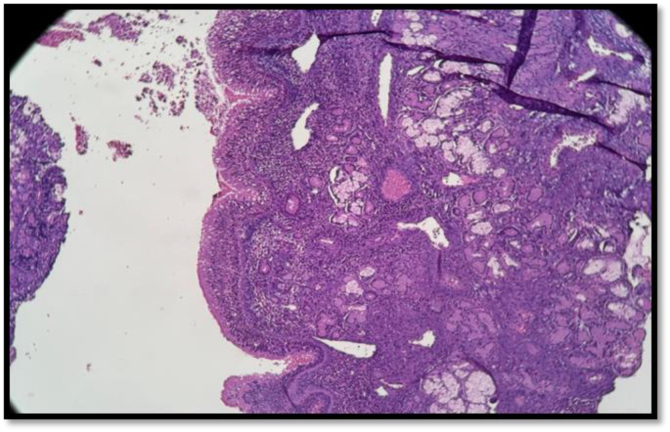
Histopathology of right maxillary sinus.

**Figure 4:**
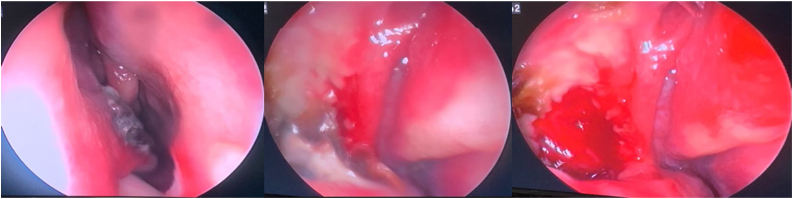
Postoperative right maxillary sinus.

Patient was provisionally diagnosed as chronic rhinosinusitis with left deviated nasal septum and underwent septoplasty with unilateral functional endoscopic sinus surgery. Septal deviation was corrected. Right side uncinectomy was done followed by right middle meatal antrostomy. Right maxillary sinus opening was widened, edematous mucosa was noted along with a mucous filled cystic sac (Fig. 2) in the right maxillary sinus. Swab was taken of the contents and sent for culture and antibiotic sensitivity and entire sac wall was sent for HPR. Postoperatively the patient was given a course of intravenous antibiotics and steroids. On histopathology (Fig. 3), the subepithelium showed focal areas of mutinous material surrounded by dense chronic inflammatory cell infiltrate, suggesting a diagnosis consistent with mucocele with chronic sinusitis. The swab culture and sensitivity were positive for *Staphylococcus epidermidis*. He was discharged on oral clarithromycin, and saline nasal wash. Patient underwent diagnostic nasal endoscopy with suction clearance (Fig. 4) on his next follow up visit. Further follow up visits showed uneventful healing at the surgical site.

## Discussion

Many theories have been suggested to explain the mechanisms responsible for the formation and growth of mucoceles, including cystic degeneration of a seromucous gland, even though the histopathological and molecular examinations showed a dynamic process of bone resorption, where inflammation takes part, through cytokines, which act between the bone and the epithelium releasing prostaglandins.^3^ These mechanisms are key players in the development of a mucocele, along with sinus ostia obstruction.

According to Yuria Ablanedo-Terrazas et al., based on a prospective study on 165 HIV infected patients, 21 patients (12.7%) presented with IRIS in the head and neck region. IRIS is usually a consequence of exaggerated activation of the immune system against antigens or pathogens manifested as an inflammatory unmasking of a previously untreated subclinical infection or as a paradoxical clinical deterioration.^4^ There is no recent literature highlighting IRIS mediated maxillary mucocele pertaining to the Indian population.

According to Michael Friedman et al., a retrospective review of HIV positive patients who underwent endoscopic sinus surgery, showed that 75% of the patients demonstrated improvement in symptoms and endoscopic evidence of infection control. Even patients with low CD4 counts (100 mL) enjoyed a significant improvement rate (67%) and a low complication rate.^5^ In our case, the patient did not present with any postoperative complications or delayed healing.

## Conclusion

IRIS is an exaggerated immune-inflammatory reaction in an HIV infected individual embarking on HAART and showing a 2–4-fold rise in CD4 counts over a short period. Thus, mucocele of the sinuses should be an expected entity in newly diagnosed HIV individuals on antiretrovirals with prior low CD4 counts (< 100 cells/mm^3^) with chronic rhinosinusitis.

## Funding

NIL.

## Conflicts of interest

The authors declare no conflicts of interest.

I express my thanks to Dr. Rajesh Havaldar, M.S., Assistant Professor, Department of Otorhinolaryngology and Head and neck surgery, J. N. Medical College for his support, constant encouragement and guidance. His immense knowledge and plentiful experience have encouraged me in all the time of my academic research and daily life.

I would like to express my gratitude to Dr. Shilpa Mallapur, Senior Resident, Department of Otorhinolaryngology and Head and neck surgery, for her constant encouragement and support during my report preparation. Her ideologies and suggestions have helped me greatly.
